# The Role of Insulin in the Proliferation and Differentiation of Bovine Muscle Satellite (Stem) Cells for Cultured Meat Production

**DOI:** 10.3390/ijms26094109

**Published:** 2025-04-25

**Authors:** Eun Ju Lee, Sibhghatulla Shaikh, Syed Sayeed Ahmad, Jeong Ho Lim, Ananda Baral, Sun Jin Hur, Jung Hoon Sohn, Inho Choi

**Affiliations:** 1 Department of Medical Biotechnology, Yeungnam University, Gyeongsan 38541, Republic of Korea; gorapadoc0315@hanmail.net (E.J.L.); sibhghat.88@gmail.com (S.S.); sayeedahmad4@gmail.com (S.S.A.); lim2249@naver.com (J.H.L.); ananbaral@gmail.com (A.B.); 2 Research Institute of Cell Culture, Yeungnam University, Gyeongsan 38541, Republic of Korea; 3 Department of Animal Science and Technology, Chung-Ang University, Anseong 17546, Republic of Korea; hursj@cau.ac.kr; 4 Synthetic Biology Research Center, Korea Research Institute of Bioscience and Biotechnology (KRIBB), 125 Gwahak-ro, Yuseong-gu, Daejeon 34141, Republic of Korea; sohn4090@kribb.re.kr

**Keywords:** muscle satellite (stem) cell, proliferation, differentiation, growth factor, insulin, cultured meat

## Abstract

Muscle satellite (stem) cells (MSCs) reside in skeletal muscle and are essential for myogenesis. Thus, MSCs are widely used in cultured meat research. This study aimed to identify substances that promote MSC proliferation and differentiation while maintaining their intrinsic properties, with the long-term goal of replacing fetal bovine serum (FBS) for bovine MSC cultivation. Therefore, this study evaluated the effects of six growth factors (TGF-β, HGF, PDGF, insulin, IGF-1, and EGF) and the cytokine IL-2 on bovine MSCs. Each factor was applied during the proliferation and differentiation of MSCs, and the proliferation rate, differentiation rate, and expression of relevant mRNA and proteins were analyzed. Insulin most effectively promoted MSC proliferation and differentiation. Specifically, insulin increased cell proliferation rates, proliferation markers Ki67 and PCNA expressions, and markers of differentiation, such as myotube formation and creatine kinase activity, alongside the expressions of MYOD, MYOG, and MYH. Furthermore, insulin suppressed low FBS-induced reductions in proliferation and differentiation markers. This study suggests insulin can promote MSC proliferation and differentiation and reduce FBS usage. Thus, this study provides a potential means of cultivating MSCs on a large scale for cultured meat production.

## 1. Introduction

Cultured meat is similar to conventional meat, but is produced by culturing livestock cells. Substantial research efforts are being expended on this emerging field to address future predicted shortages in animal-derived food products, resolve environmental pollution issues associated with livestock farming, or develop technologies that provide hygienic, healthy animal protein without requiring animals to be slaughtered [1,2].

The production of products is similar to skeletal muscle (or meat) from animal cells, which represents the primary challenge in cultured meat production. Skeletal muscle satellite (stem) cells (MSCs) are the most commonly used cells in cultured meat research [3,4,5]. MSCs are located between the sarcolemma and the basal lamina of muscle fibers. They usually remain quiescent but become activated in response to external stimuli and then undergo proliferation and differentiation to form multinucleate cells (muscle fibers) due to the fusion of hundreds or thousands of cells under the direction of muscle regulatory factors (MRFs), growth factors, and cytokines. The entire process is called myogenesis [6,7,8]. MSCs involved in myogenesis express the transcription factors Pax3 and Pax7 and the sequential expressions of MRFs, such as Mrf4, Mrf5, MyoD, and myogenin (MYOG), which regulate myogenesis [9,10,11]. In addition, extracellular matrix (ECM) proteins (fibromodulin, matrix gla protein, and dermatopontin) and certain membrane proteins (IgLON4 and IgLON5) regulate myogenesis [12,13,14].

MSCs isolated from livestock skeletal muscle proliferate when cultured in vitro, and a reduction in the concentration of FBS in the medium upon reaching the growth plateau causes the cells to differentiate into myotubes, which resemble muscle fibers [15,16]. This method of culturing MSCs is a fundamental aspect of cultured meat production. However, similar to other primary cells, MSCs have long-term and large-scale shortcomings because their characteristics are difficult to maintain after several passages [17,18]. Furthermore, MSCs have significantly lower proliferation and differentiation efficiencies in culture media that do not contain FBS. Since FBS is extremely expensive and derived from livestock, researchers in the cultured meat production field strive to produce cultured meat without FBS [19].

The culture medium is a major cost factor in cultured meat production and accounts for over 95% of the total costs in cultured meat production, for which FBS is largely responsible [20]. Serum is a complex mixture of essential nutrients, growth factors, metabolites, and hormones essential for cell adhesion, proliferation, and differentiation in animal cell culture systems [21]. However, despite its usefulness, the use of serum for cultured meat production poses challenges due to batch variability and cost issues, potential biological toxicity, and ethical concerns related to serum production and animal welfare [22,23]. Therefore, developing alternatives to FBS is a prerequisite for successfully commercializing cultured meat production technologies.

To achieve this objective, this study aimed to identify the growth factors and cytokines involved in the proliferation and differentiation of bovine MSCs [24] and to apply them to cultured meat production. The growth factors and cytokines used in this study were selected based on existing research findings, ease of availability, and low toxicity. The effects of growth factors, (Transforming Growth Factor-beta 1: TGF-β1 [25,26], Hepatocyte Growth Factor: HGF [27], Platelet-Derived Growth Factor: PDGF [28,29], insulin [30,31,32], Insulin-like growth factor 1: IGF-1 [33,34], Epidermal Growth Factor: EGF [35], and cytokine Interleukin 2: IL-2 [36]), were investigated, which have previously been shown to influence growth and differentiation in human and mouse studies.

## 2. Results

### 2.1. The Differentiation Ability of Bovine MSCs

**Figure 1:**
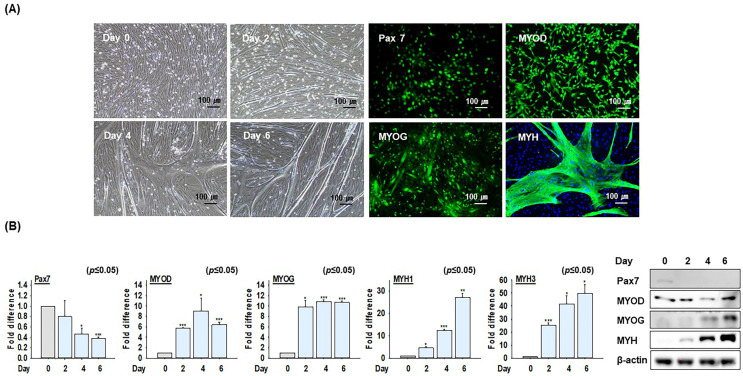
Bovine MSC differentiation. When cells were at 100% confluency, growth media (20% FBS) was switched to differentiation media (2% FBS) and cells were incubated for a further 0, 2, 4, or 6 days. (**A**) Cell morphology and protein Pax7 (green); after 3 days in growth medium, MYOD (green); after 2 days in differentiation medium, MYOG (green); after 4 days in differentiation medium, MYH (green; after 6 days in differentiation medium, blue; nuclei), expression as determined by immunocytochemistry. (**B**) mRNA and protein expressions of Pax7, MYOD, MYOG, MYH1, and MYH3 as determined by real-time RT-PCR and Western blot, respectively. Results are presented as the mean ± SD (*n* > 3), * *p* ≤ 0.05, ** *p* ≤ 0.001, *** *p* ≤ 0.0001.

The growth medium was switched to a differentiation medium when bovine MSCs reached confluency in culture dishes, and cells were cultured for 0, 2, 4, and 6 days. At each time point, the expressions of MSC marker genes such as Pax7 (green) and proteins expressed explicitly during MSC differentiation, including MYOD (green), MYOG (green), and myosin heavy chain (MYH, green), were subjected to immunocytochemistry, which showed that myotube formation increased as MSC differentiation progressed. Most cells expressed Pax7 before differentiation, and the expressions of specific marker proteins were observed at each differentiation stage (Figure 1A). In addition, the mRNA and protein expression levels of differentiation-related marker genes (Pax7, MYOD, MYOG, MYH1, and MYH3) were monitored after differentiation for 0, 2, 4, or 6 days using real-time RT-PCR and Western blot, respectively. These findings confirmed that the expressions of marker genes involved in MSC differentiation increased during differentiation (Figure 1B). These results indicate that the MSCs used in these experiments were of high purity and that their gene (mRNA) expression patterns accurately reflected the differentiation process.

### 2.2. Screening of Growth Factors and Cytokine That Promote MSC Proliferation and Differentiation

**Figure 2:**
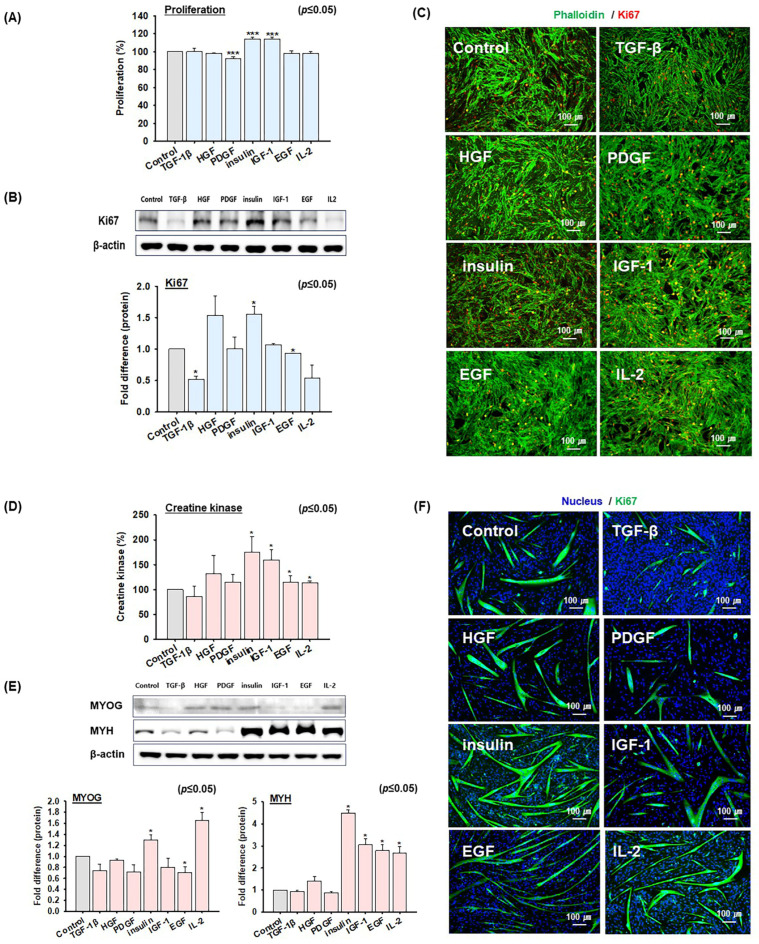
Bovine MSC proliferation and differentiation after treatment with growth factors or IL2. MSCs were cultured in a growth medium (20% FBS) supplemented with or without growth factors (recombinant human TGF-β, HGF, PDGF, insulin, IGF-1, or EGF) and a cytokine (IL-2) for 4 days. (**A**) MCS proliferation was determined using the MTS assay. (**B**) Ki67 protein expression by Western blot analysis, band intensities were evaluated using Image J software (Version 1.54). (**C**) Ki67 expression by immunocytochemistry (red; Ki67, green; phalloidin). When MSCs reached 100% confluency, growth media was switched to differentiation media (2% FBS) supplemented with or without growth factors and incubated for 2 days. (**D**) MSC differentiation as determined by creatine kinase activity. (**E**) MYOG and MYH protein expressions by Western blot and band intensities using image J. (**F**) MYH expression by immunocytochemistry (green; MYH, blue; nuclei). Results are presented as the mean ± SD (*n* > 3), * *p* ≤ 0.05, *** *p* ≤ 0.0001.

Six different human recombinant growth factors (TGF-1β, HGF, PDGF, insulin, IGF-1, and EGF) and one human recombinant cytokine (IL-2) were screened to check the effects on MSC proliferation and differentiation. The concentrations were determined as previously described [37,38,39,40,41,42,43]. MSCs were cultured in growth medium with or without these individual growth factors or IL-2 for 4 days. MSC proliferation was analyzed using the MTS assay after culturing cells under the above-mentioned conditions. MSC proliferation was significantly increased by insulin (114 ± 2%), bp-insulin (insulin was isolated from bovine pancreas, 107 ± 1%), and IGF-1 (114 ± 2%) compared to untreated cells (100%), while PDGF (92 ± 2%) treatment decreased proliferation slightly. Other growth factors had no significant effects (Figure 2A and Supplementary Figure S1A). Western blot and staining analyses of the Ki67 protein expression (cell proliferation maker, red) [44] showed that HGF and insulin treatment increased Ki67 expression, while TGF-β, PDGF, EGF, and IL-2 had the inverse effect. The increase in Ki67, a proliferation-related marker, indicates enhanced cell proliferation, with insulin treatment resulting in the most significant effect (Figure 2B,C, Supplementary Figure S1B).

After reaching cell confluency in the proliferation phase, MSCs were cultured in a differentiation medium containing the individual growth factors or IL-2 for 2 days. Creatine kinase activity, an indicator of the MSC differentiation rate, was used to the quantify degrees of differentiation in similarly differentiated cells. Myotube formation and creatine kinase activities were elevated in cells treated with insulin (175 ± 31%), IGF-1 (160 ± 20%), EGF (115 ± 13%), or IL-2 (114 ± 3%) compared to untreated cells (100%), which indicated that insulin and IGF-1 had the most pronounced effects on MSC differentiation. Conversely, TGF-1β (86 ± 21%) resulted in a decrease in differentiation rate (Figure 2D and Supplementary Figure S1C). Additionally, Western blot analysis of the differentiation markers MYOG and MYH, which are known to be upregulated during the later stages of muscle differentiation, revealed that insulin and IL-2 treatment significantly increased MYOG expression. In contrast, insulin, IGF-1, EGF, and IL-2 increased MYH expression (Figure 2E). The immunocytochemical analysis revealed enhanced MYH expression in cells treated with HGF, insulin, IGF-1, EGF, and IL-2. The strong expression of MYH protein suggests an increased level of differentiation in MSCs (Figure 2F). In addition, the effects on MSC proliferation were analyzed after combining the most effective agents, insulin and IGF-1. Bovine MSCs were cultured in a proliferation medium supplemented with insulin, IGF-1, or insulin + IGF-1 for 4 days and then analyzed using the MTS assay. The results showed that the proliferation rate of cells treated with insulin + IGF-1 (117 ± 2%) was not significantly different from the cells treated with insulin alone (116 ± 3%) or IGF-1 alone (111 ± 1%) (Supplementary Figure S2A,B). Subsequently, cells were cultured in a differentiation medium supplemented with insulin, IGF-1, or insulin + IGF-1 for 2 days during differentiation to check the effects of insulin and IGF-1. Differentiation rates were assessed using the creatine kinase activity culture for two days. The differentiation rates of cells treated with insulin (125 ± 1%) or IGF-1 (124 ± 4%) did not significantly increase compared to cells treated with insulin + IGF-1 (128 ± 6%) (Supplementary Figure S2C,D). These findings indicated that insulin can substantially increase MSC proliferation and differentiation more than the other examined biomolecules.

### 2.3. Effects of Insulin on MSC Proliferation and Differentiation

**Figure 3:**
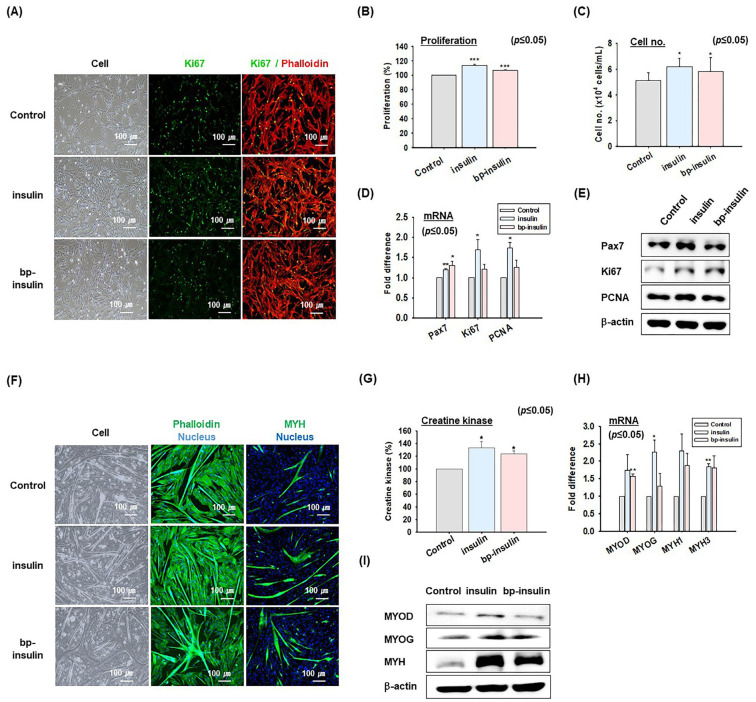
The effects of insulin on MSC proliferation and differentiation. MSCs were cultured in a growth medium (20% FBS) for 4 days supplemented with or without human recombinant insulin or bovine pancreatic insulin (bp-insulin). (**A**) Cell morphologies and Ki67 expression as determined by immunocytochemistry (red phalloidin, green Ki67). (**B**) MSC proliferation by MTS assay. (**C**) Cell number counting. (**D**) Pax7, Ki67, and PCNA mRNA expression determined by real-time RT-PCR. (**E**) Western blot analysis of Pax7, Ki67, and PCNA protein expressions. When MSCs reached 100% confluency, the growth medium was switched to a differentiation medium (2% FBS) supplemented with or without human recombinant insulin or bp-insulin and incubated for 2 days. (**F**) Cell morphology of phalloidin (green; Phalloidin, blue: nuclei), or MYH (green; MYH, blue; nuclei) expression by immunocytochemistry. (**G**) MCS differentiation assessed by creatine kinase activity. (**H**) MYOD, MYOG, MYH1, and MYH3 mRNA expressions by real-time RT-PCR. (**I**) Western blot analysis of MYOD, MYOG, and MYH protein expressions. Results are presented as the mean ± SD (*n* > 3), * *p* ≤ 0.05, ** *p* ≤ 0.001, *** *p* ≤ 0.0001.

Bovine MSCs were cultured in a growth medium supplemented with human recombinant insulin or bp-insulin (bovine pancreatic insulin, insulin isolated from bovine pancreas) for 4 days. Proliferation rates were determined using the MTS assay and cell number counting, and the expression of related genes mRNA and proteins were analyzed by real-time RT-PCR and Western blotting, respectively. Immunocytochemical analysis of Ki67 protein expression revealed that insulin treatment increased nuclear Ki67 expression (Figure 3A). MSCs grown in media supplemented with insulin (114 ± 2%, cell number: 7.63 × 10^4^ cells/mL) or bp-insulin (107 ± 1%, cell number: 7.27 × 10^4^ cells/mL) proliferated faster than untreated controls (100%, cell number: 4.87 × 10^4^ cells/mL, Figure 3B,C). Both insulin and bp-insulin significantly increased the mRNA and protein levels of Ki67, a cell proliferation marker, while insulin increased the mRNA and protein levels of Pax7. In addition, insulin significantly increased PCNA expression, a cell proliferation marker specifically expressed in MSCs [45] (Figure 3D,E).

MSCs were cultured for 2 days in differentiation media supplemented with insulin or bp-insulin to investigate the effect of insulin on the differentiation rate. The results indicate that insulin and bp-insulin promote myotube formation and increase MYH protein expression, as determined by immunocytochemistry (Figure 3F). Moreover, creatine kinase activity increased in MSCs treated with insulin (137 ± 13%) or bp-insulin (126 ± 5%) compared to untreated cells (100%) (Figure 3G). Furthermore, insulin and bp-insulin both increased the expressions of the differentiation marker genes MYOD, MYOG, MYH1, and MYH3 (Figure 3H,I). These results suggest that insulin plays a crucial role in the proliferation and differentiation of bovine MSCs.

### 2.4. Effect of Insulin on MSC Proliferation and Differentiation at Different FBS Concentrations

**Figure 4:**
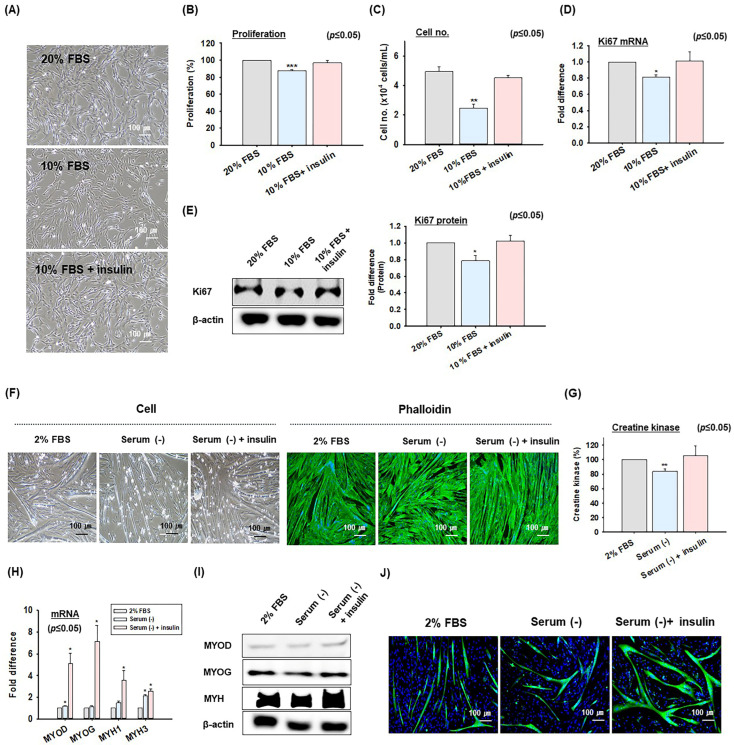
The effects of insulin on MSC proliferation and differentiation at different FBS concentrations. MSCs were cultured in growth media (20% or 10% FBS) supplemented with or without recombinant human insulin for 4 days. (**A**) Cell morphologies. (**B**) MCS proliferation was determined using the MTS assay. (**C**) Cell number counting. (**D**) Ki67, mRNA expression by real-time RT-PCR. (**E**) Ki67 protein expression by Western blot and band intensities using image J. When MSCs reached 100% confluency, growth media was switched to differentiation media [2%FBS or serum-free media (serum (−))] supplemented with or without recombinant human insulin and incubated for 2 days. (**F**) Cell morphology and phalloidin staining (green; Phalloidin, blue; nuclei) (**G**) MSC differentiation as determined by creatine kinase activity. (**H**) MYOD, MYOG, MYH1, and MYH3 mRNA expressions as determined by real-time RT-PCR. (**I**) MYOD, MYOG, and MYH protein expressions determined by Western blot. (**J**) Immunocytohemistry analysis of MYH expression (green; MYH, blue; nuclei). Controls were cells incubated with 20% FBS or 2% FBS. Results are presented as the mean ± SD (*n* > 3), * *p* ≤ 0.05, ** *p* ≤ 0.001, *** *p* ≤ 0.0001.

The effect of insulin on MSC proliferation and differentiation was also investigated using different FBS concentrations in growth or differentiation media (Figure 4). First, MSCs were cultured for 4 days in proliferation media containing 20% FBS, proliferation media containing 10% FBS, or proliferation media containing 10% FBS supplemented with insulin (10 μg/mL). Reducing the FBS concentration percentage from 20% to 10% decreased MSC numbers but adding insulin to the 10% FBS medium increased MSC numbers to levels comparable with the 20% FBS medium (Figure 4A). Additionally, MTS assays and cell number counting showed that the proliferation rate of cells cultured in 10% FBS was ~13% lower than that of cells cultured in 20% FBS, whereas cells cultured in insulin supplemented with 10% FBS had a proliferation rate similar to those cultured in 20% FBS (Figure 4B,C). Real-time RT-PCR and Western blot analyses for Ki67 revealed that MSCs cultured in 10% FBS expressed Ki67 at lower levels than MSCs cultured in 20% FBS, while MSCs cultured in insulin supplemented medium with 10% FBS had Ki67 expression levels similar to those of cells cultured in 20% FBS (Figure 4D,E).

MSC differentiation was also assessed using a differentiation medium containing 2% FBS, serum-free differentiation media [Serum (−)], and serum-free differentiation media supplemented with insulin [Serum (−) + insulin]. Myotube formation was less in the serum-free medium compared to the medium containing 2% FBS. On the other hand, adding insulin to serum-free medium resulted in myotube formation levels similar to those for the 2% FBS medium (Figure 4F). In the analogous experiments, cells cultured in a serum-free medium had a creatine kinase activity level of 84 ± 3% compared to cells cultured in the 2% FBS (100%). In contrast, cells cultured in a serum-free medium supplemented with insulin had a creatine kinase activity level similar to those cells cultured in the 2% FBS medium (Figure 4G). Moreover, the mRNA expressions of MYOD, MYOG, MYH1, and MYH3 were higher in the serum-free media than in the 2% FBS media, and adding insulin to the serum-free media further increased these expressions higher than those of the serum-free media alone (Figure 4H,I). Furthermore, the immunocytochemistry analysis illustrated reduced MYH expressions the serum-free mediums compared with those with 2% FBS. However, serum-free media supplemented with insulin showed increased MYH expression (Figure 4J). These findings suggest that insulin can partially substitute the role of FBS in MSC proliferation and differentiation.

## 3. Discussion

Researchers, companies, and even countries continue to progress toward commercializing cultured meat in the hope that such products will provide humanity with sustainable and high-quality animal protein [46]. However, these entities must first overcome several technical hurdles associated with mass producing cultured meat and reduce production costs. Without comprehensively addressing these issues, the commercialization of cultured meat will likely remain theoretical, regardless of country-specific regulations related producing and selling cultured meat.

Currently, skeletal muscle proliferation and differentiation extend beyond medical applications to cultured meat production. Myogenesis converts cells into edible meat and is central to cultured meat production. The basic concept of cultured meat production is similar to the methods used in biopharmaceutical manufacturing processes. Initially, a small number of cells are cultured and gradually expanded through several stages to produce extensive cell numbers. Subsequently, these cells are differentiated into forms that resemble muscle cells or tissues to create products similar to meat or meat ingredients [47,48]. Therefore, the following conditions are required for the commercial production of cultured meat. First, the cells must maintain their characteristics, continue proliferating after extensive passaging, and retain the ability to differentiate into muscle cell-like types. Second, the production costs required for long-term, large-scale culturing must be reduced. Third, the materials used for the large-scale production of cultured meat must be safe for human consumption. Thus, this study aimed to conduct fundamental research to facilitate the development of cultured meat production technologies through the mass culture of MSCs. This includes promoting the proliferation and differentiation of MSCs and minimizing the use of FBS, which currently constitutes a significant portion of cell culture production costs, or developing serum-free media that do not require FBS. This latter objective involves identifying growth factors and cytokines that support cultured meat production.

MSCs are the cells most commonly used in cultured meat production. However, similar to other primary cells, MSCs tend to lose their characteristics and abilities to proliferate and differentiate with increased passages, which suggests these cells are unsuitable for future cultured meat production [49]. Subsequently, efforts have been made to address these problems by implementing spontaneously immortalized cells or modifying MSC genes [50,51]. However, these cells also face challenges related to tumorigenicity and concerns regarding the use of genetically modified organisms (GMOs), which make them less than ideal for cultured meat production. Some researchers have explored using pluripotent embryonic stem cells for cultured meat production [52]; however, while this approach is theoretically possible, it requires multiple cell culture stages, further complicating the production process.

Research on MSCs has primarily focused on human or mouse models or cell culture research models predominantly employing the C2C12 cell line derived from mice or the L6 cell line derived from rats. Therefore, systematic research on livestock, is limited, particularly cattle, pigs, and chicken MSCs. Our research team has previously studied the characteristics of bovine MSCs during proliferation and differentiation with particular emphasis on the identification and functional annotation of genes uniquely expressed during these processes [53,54]. The cells used in the present study were isolated from bovine skeletal muscle and used to identify growth factors or cytokines that promote the proliferation and differentiation of bovine MSCs, investigate the effects of these biomolecules on bovine MSC proliferation and differentiation, and provide scientific evidence as to whether these factors can replace FBS in proliferation and differentiation media.

To this end, we investigated the effects of six recombinant human growth factors (TGF-β, HGF, PDGF, insulin, IGF-1, and EGF) and a cytokine (IL-2), which have been previously reported to influence MSC proliferation or differentiation (Figure 2). The results showed that in terms of cell proliferation, insulin and IGF-1 were the most effective, as evidenced by the expression of the proliferation marker protein Ki67 and cell proliferation rates. Regarding differentiation, assessed through myotube formation, the expressions of myogenic marker genes, such as MYH, creatine kinase activity, insulin, and IGF-1, were found to have the greatest effects. Interestingly, despite being from the same species as bovine MSCs, bp-insulin did not perform as effectively as human insulin (Figure 3). Furthermore, it was interesting that no synergistic or additive effects were observed when insulin and IGF-1 were added simultaneously during MSC proliferation and differentiation (Supplementary Figure S2). Consequently, for subsequent experiments, we focused on insulin.

This comparative study on the effects of insulin and bp-insulin on MSC proliferation and differentiation showed that insulin was more effective than bp-insulin at promoting proliferation, as evidenced by Ki67 immunocytochemistry and the MTS assay. Insulin promoted Ki67 expression and enhanced the mRNA and protein expressions of other proliferation markers, such as PCNA and the MSC marker gene Pax7 (Figure 3A–E;). Furthermore, differentiation experiments showed that insulin and bp-insulin positively affected myotube formation and creatine kinase activity. Insulin and bp-insulin also increased the mRNA and protein expression of the MYOD and MYOG genes involved in the early and late stages of MSC differentiation and those of genes associated with the myofibril structure of muscle fibers (MYH1 and MYH3) (Figure 3F–I).

Based on the observed positive effects of insulin on MSC proliferation and differentiation, we investigated whether insulin could replace FBS during the proliferation and differentiation of MSCs. Researchers generally use media containing 20% FBS for MSC proliferation and 2% for differentiation [55,56]. Notably, cell proliferation experiments showed that decreasing FBS from 20% to 10% in a proliferation medium reduced MSC proliferation. However, when insulin was added to a medium containing 10% FBS, cell proliferation was similar to that observed for a 20% FBS containing medium. These findings were supported by the cell proliferation rate analysis and Ki67 gene expression results (Figure 4A–E). Furthermore, removing of 2% FBS from the differentiation medium in the MSC differentiation studies reduced the myotube width and creatine kinase activity. However, when insulin was added to the FBS-free medium, the extent of MSC differentiation was similar to that observed for the 2% FBS-containing medium. Moreover, insulin treatment increased the expressions of marker genes related to MSC differentiation more than with 2% FBS (Figure 4F–J). These results suggest that insulin alone can partially replace FBS during MSC proliferation and differentiation. However, the potential for insulin treatment to partially replace FBS in the proliferation and differentiation of MSCs suggests the need for further mechanistic studies and research into its application in cultured meat production processes. Additionally, to address the deficiencies caused by removing of FBS, an analysis of the FBS components and research on the combination of insulin and other growth actors is required. Currently, the authors are conducting further research to analyze these factors.

The proliferation and differentiation of MSCs at a low cost and on a large scale is critical for the commercial production of cultured meat [57]; thus, a cell culture media that can mimic the long-term characteristics of MSCs and enable these cells to differentiate into myofiber-like cells is critical. Hence, given the accepted importance of FBS for cultured meat production, growth factors and cytokines can be reasonably considered candidate replacement substances [55,58]. The present study focused on growth factors and IL-2, since these influence MSC proliferation and differentiation primarily in human or mouse models [59,60,61,62,63]. Numerous studies have demonstrated that IGF-1 promotes MSC proliferation and differentiation and can be used for cultured meat [64,65,66]. However, our results showed that insulin exhibited increased potency over MSC proliferation and differentiation, and thus, insulin formed the primary focus of this study. It is also important to note that FGF-2 is included in proliferation media to prevent MSCs from prematurely entering the differentiation stage from the proliferation phase.

Insulin plays essential roles in skeletal muscle glucose uptake and metabolism [67] and promotes cell proliferation and protein synthesis through signaling pathways involving Shc and MAP kinases, such as MEK and ERK [68,69]. In our study, insulin promoted the proliferation of bovine MSCs, as evidenced by increased expressions of proliferation markers such as Ki67 and PCNA. Given that Pax7 is a transcription factor essential for muscle regeneration and the normal function of MSCs [70], it was interesting to find that insulin increased the expression of the MSC marker gene Pax7. This observation indicates that insulin promotes MSC proliferation and enhances Pax7 expression and thereby supporting the normal proliferation and differentiation of MSCs. Thus, while most growth factors promote MSC proliferation and inhibit differentiation, insulin and IGF-1 act alternatively by promoting the MSC proliferation and differentiation [71,72,73]. Furthermore, both growth factors activate the PI3 kinase-Akt and Ras/Raf/p42/p44-MAPK signaling pathways, promoting the expressions of the muscle differentiation-related genes MYOD and MYOG [71,72]. This explains the absence of any additive or synergistic effects when insulin and IGF-1 were co-administered to MSCs. Furthermore, although our study demonstrates that insulin positively affects MuSC proliferation and differentiation, more research is needed to determine whether these effects apply to long-term, large-scale production processes.

Various cellular and molecular mechanisms underlying MSC proliferation and differentiation have been elucidated [24,74]. Nevertheless, to maximize the potential use of MSCs for cultured meat production, identifying substances that can stimulate pathways other than those involved in MSC proliferation and differentiation remains essential. In this regard, research remains on identifying substances that promote MSC proliferation or differentiation [15,74] to help maintain MSC characteristics when combined with insulin. Additionally, to perform large-scale cultured meat production, MSCs must be passaged multiple times over an extended period before being allowed to differentiate. Meanwhile, recent significant serum-free media developments involving fetuin, BSA, insulin, transferring, selenium, and FGF-2 have been published [75] In summary, the present study demonstrates that insulin has a remarkably positive effect on the proliferation and differentiation of bovine MSCs and can partially replace FBS in MSC culture media. We are confident that these findings provide crucial information for future research on commercialized cultured meat production using MSCs.

## 4. Materials and Methods

### 4.1. Bovine MSC Isolation

Isolation of MSCs was performed as previously described [15]. Immunocytochemistry was conducted to assess the Pax7 protein expression and verify the purity of the isolated MSCs. This analysis revealed that 92% of the cells were Pax7 positive (Pax7 positive cells/total nuclei × 100, Supplementary Figure S3). All experiments were performed in accordance with the guidelines provided by the Institutional Animal Care and Use Committee of Yeungnam University (AEC2022-022).

### 4.2. Bovine MSC Proliferation and Differentiation

Bovine MSCs were cultured in growth media [Ham’s F10 (F-10 Nutrient Mixture, Cytiva, Marlborough, MA, USA) + 20% or 10% FBS (Fetal bovine serum, Cytiva) + 1% P.S (Penicillin-streptomycin, Thermo Fisher Scientific, Waltham, MA, USA) + 5 ng/mL fibroblast growth factor-2 (FGF-2, Miltenyi Biotec, Auburn, CA, USA)]. Cells were incubated in DMEM + 2% FBS or serum-free + 1% P.S (differentiation media) for 2 days to induce myogenic differentiation.

### 4.3. Growth Factor or Cytokine Treatment

Seven growth factors [insulin, bp-insulin (Sigma Aldrich, St. Louis, MO, USA), TGF-β, HGF, PDGF, IGF-1, or EGF, (Miltenyi Biotec)] and IL-2 (Miltenyi Biotec) were treated at different concentrations (Supplementary Table S1).

### 4.4. Counting MSCs

MSCs were cultured in growth media supplemented with or without insulin or bp-insulin for 4 days. After incubation, the MSCs were dissociated using trypsin-EDTA (Thermo Fisher Scientific) and stained with a green/red cell viability stain solution (Thermo Fisher Scientific). Then, the viability of the stained cells was measured using the Countess 3 FL (Thermo Fisher Scientific).

### 4.5. MTS Analysis

Cells were cultured in growth media supplemented with CellTiter 96 Aqueous One Solution Reagent (Promega, WI, USA) in a humidified 5% CO_2_ incubator at 37 °C for 2 h. Absorbances were measured at 490 nm using a Versa max microplate reader (Tecan Group Ltd., Mannedorf, Switzerland).

### 4.6. Creatine Kinase Activity

Creatine kinase activity was measured to evaluate MSC differentiation [76]. Cells were washed with PBS, collected, and centrifuged at 1000× *g* for 15 min. Supernatants were analyzed using a creatine kinase assay kit (BioAssay Systems, Hayward, CA, USA) by a Versa max microplate reader at 340 nm and the following equation.

$$\mathrm{CK}\;(U/L)=\frac{O.D40\;\min-O.D20\;min}{O.DCALIBRATOR-O.DH_{2}O}\;\times \;150$$

### 4.7. cDNA Synthesis and Real-Time RT-PCR

cDNA synthesis and real-time RT-PCR were performed as previously described [74]. Information on the primer sequence is provided in Supplementary Table S2.

### 4.8. Western Blot

Cell proteins (100 µg) were electrophoresed and transferred to PVDF membranes. The membranes were then incubated in blocking reagent and treated with the indicated primary antibody [Pax7 (1:500), Ki67 (1:500), PCNA (Proliferating cell nuclear antigen, 1:500) MYOD (1:500), MYOG (1:1000), MYH (1:250), or β-actin (1:1000) (Santa Cruz Biotechnology, Paso Robles, CA, USA)] in 1% skim milk or BSA in Tris-buffered saline at 4 °C overnight. After washing, membranes were incubated with goat-rabbit or mouse-horseradish peroxidase (HRP)-conjugated secondary antibodies (Santa Cruz Biotechnology) at room temperature for 2 h. Blots were visualized using Super Signal West Pico Chemiluminescent Substrate (Thermo Fisher Scientific).

### 4.9. Phalloidin Staining

Phalloidin was used to selectively stain F-actin [77,78]. After removing the media, cells were washed with PBS, fixed with 10% formaldehyde (Sigma Aldrich), incubated with 0.2% Triton X-100 for 5 min, and washed with PBS cells. Cells were then stained with phalloidin (1:400, Invitrogen, Carlsbad, CA, USA), and nuclei were stained using DAPI (Sigma-Aldrich). Images were captured using a fluorescence microscope equipped with a digital camera (Nikon, Tokyo, Japan).

### 4.10. Immunocytochemistry

Immunocytochemistry was conducted, as previously described [79]. Briefly, cells were fixed with 10% formaldehyde, incubated in 0.2% Triton X-100 for 5 min, rinsed with PBS, and incubated in 1% normal goat serum for 30 min. Cells were incubated with specific antibodies [Pax7, Ki67, MYOD, MYOG, or MYH (1:50)] at 4 °C overnight and then with Alexa Fluor 488 or 594 goat anti-mouse and rabbit secondary antibodies (1:100; Thermo Fisher Scientific) at room temperature for 1 h. Cells were then stained with DAPI (Sigma-Aldrich) and imaged under a fluorescent microscope (Nikon).

### 4.11. Statistical Analysis

Tukey’s range test was used to determine the significance of differences between mean normalized gene and protein expressions. GAPDH (gene expression) or β-actin (protein expression) were used as internal controls, and the analysis was performed using one-way analysis of variance (ANOVA) in SAS ver. 9.0 (SAS Institute, Cary, NC, USA). Statistical significance was accepted for *p* values < 0.05 *, <0.001 **, and <0.0001 ***.

## Data Availability

Data will be made available on request.

## Supplementary Materials

The following supporting information can be downloaded at: https://www.mdpi.com/article/10.3390/ijms26094109/s1.
