# Supplementary material for: The Role of Insulin in the Proliferation and Differentiation of Bovine Muscle Satellite (Stem) Cells for Cultured Meat Production
Source: Int J Mol Sci. 2025 Apr 25;26(9):4109. doi: 10.3390/ijms26094109 (PMC12071896; doi:10.3390/ijms26094109)

Supplementary Data

Supplementary Table S1

Species	Growth factors	Final concentration	Reference
Human	TGF-β	5 ng/mL	Lara Pferdehirt et al., 2022
Human	HGF	5 ng/mL	Zeng et al., 2022, Miller et al., 2000
Human	PDGF	2 ng/ml	Clunn et al., 1997
Human	Insulin	10 ug/mL	Starkey et al., 2011
Human	IGF-1	100 ng/mL	Machid et al., 2003
Human	EGF	100 ng/ml	Wang et al., 2019
Human	IL2	100 ng/ml	O'Leary et al., 2017
Bovine	Insulin	10 ug/mL	Starkey et al., 2011

Supplementary Table S2

Gene	Product size (bp)	Tm (℃)	Sequence (F)	Sequence (R)
GAPDH	211	59	5'-GGGTCATCTCTGCACCT-3'	5'-ACAGTCTTCTGGGTGGCAGT-3'
Pax7	228	59	5'-GGACGTGGAGAAAAAGATCG-3'	5'-ATGCTGTGCTTGGCTTTCTT-3'
Ki67	150	59	5'-AGACCCCAGATCACACCAAG-3'	GGTCTTCCTGCATGTCCACT-3'
PCNA	209	59	5'-TGTGCTGGCAATGAAGACAT-3'	TCTCGGCATATACGTGCAAA-3'
MYOD	229	59	5'-GATGACCCGTGTTTCGACTC-3'	5'-TAGTCGTCTTGCGTTTGCAC-3'
MYOG	197	59	5'-TGGGCGTGTAAGGTGTGTAA-3'	5'-TGCAGGCGCTCTATGTACTG-3'
MYH1	235	59	5'-GGAGATGCGAGATGAAAAGC-3'	5'-CATGTTGGCCATTTCCTTCT-3'
MYH3	221	59	5'-GTACGTTACCAAGGGCCAGA-3'	5'-CGTTGGTGAAGTTGATGCAC-3'

Supplementary Figure S1

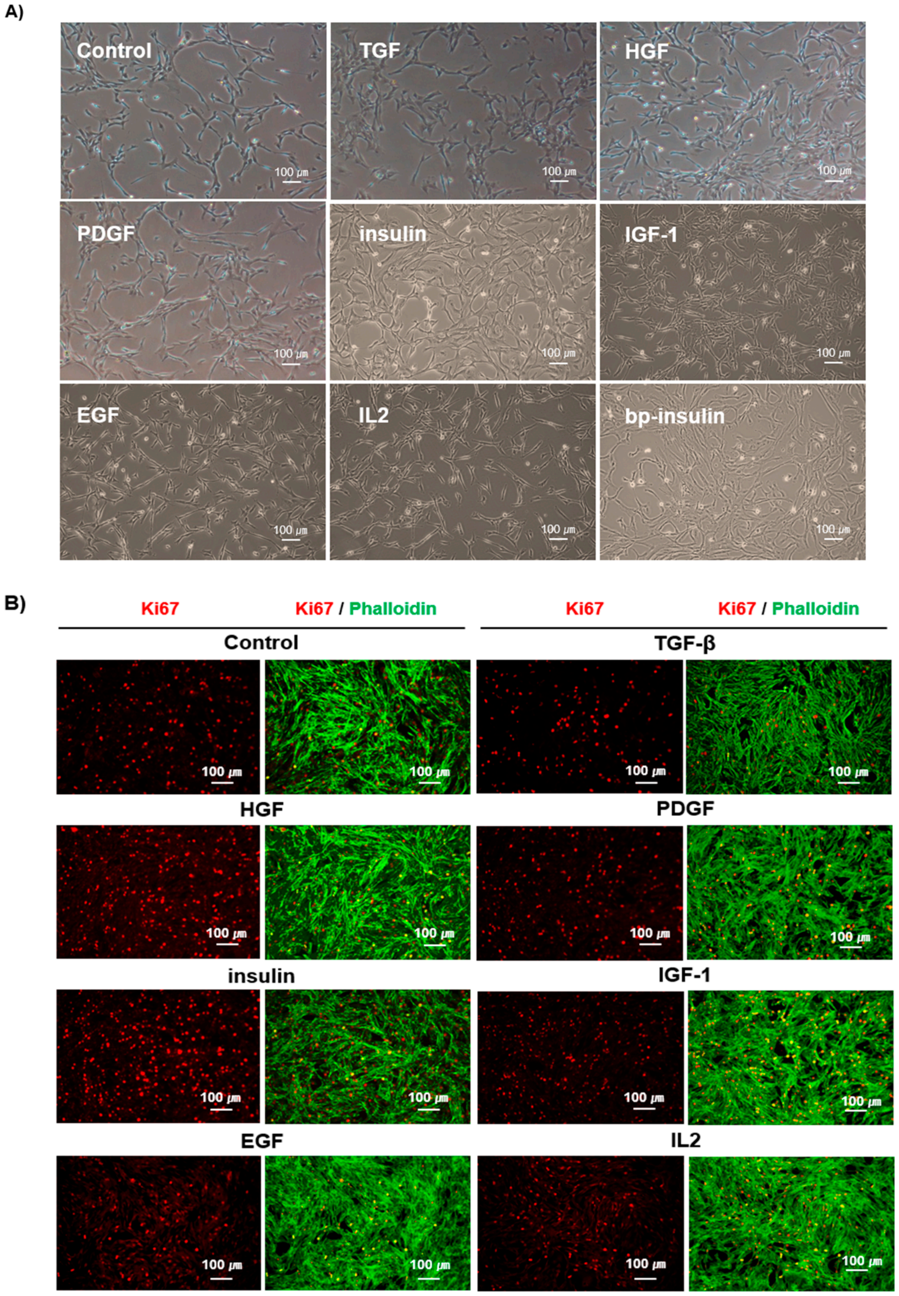

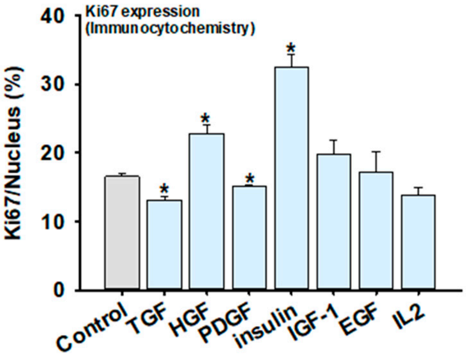

C)

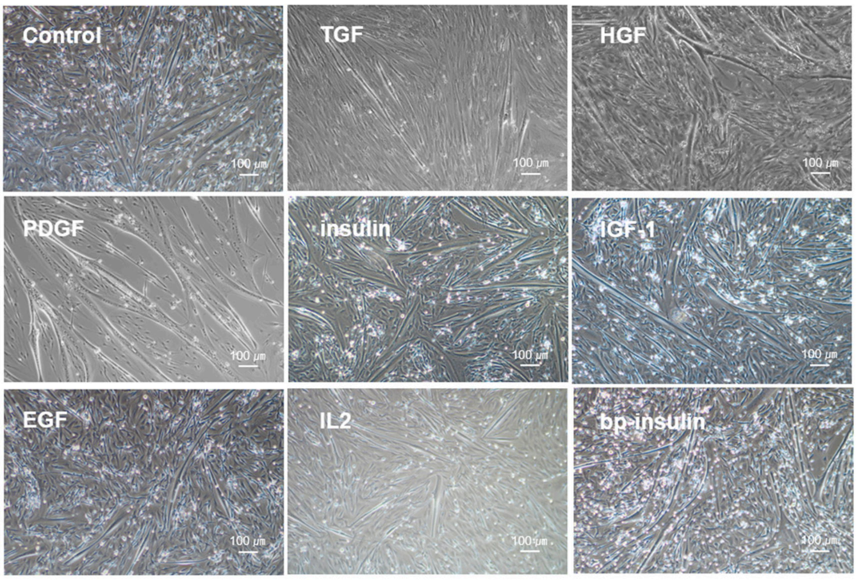

Supplementary Figure S2

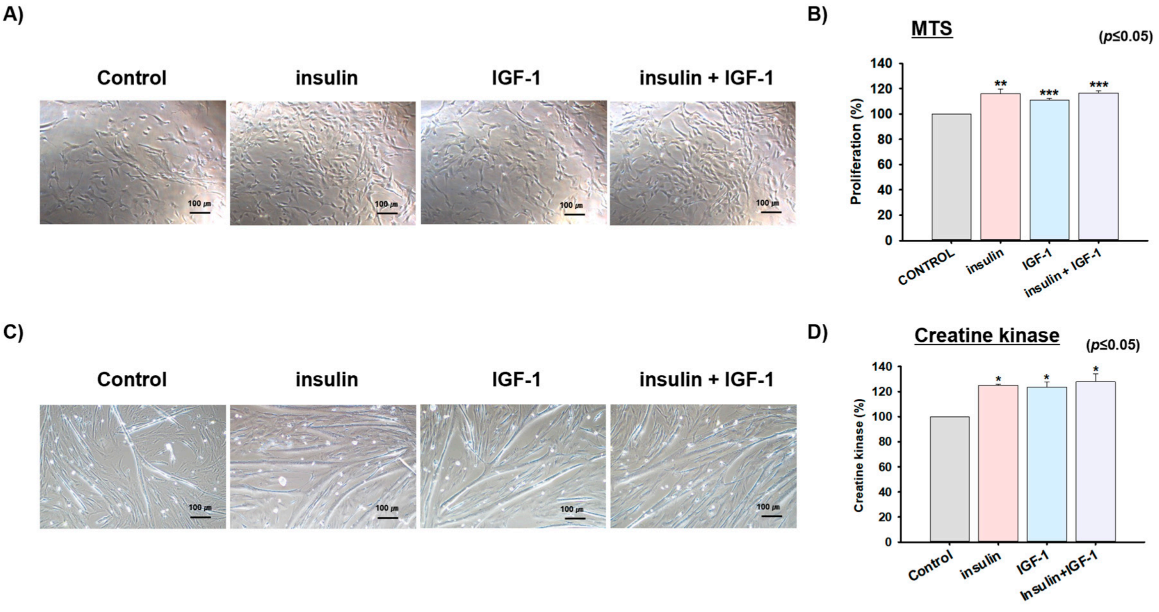

Supplementary Figure S3

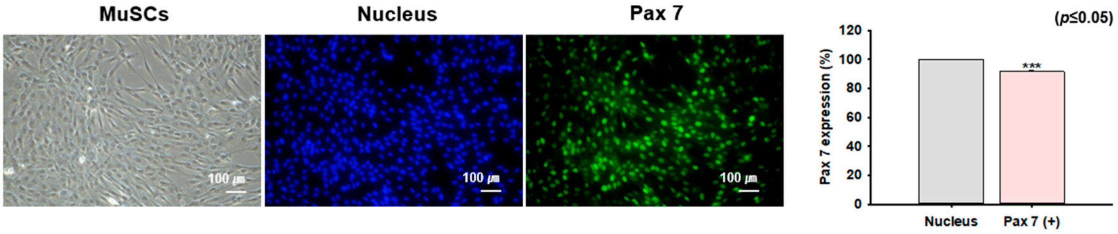

Supplement: Supplementary file 1 [file ijms-26-04109-s001.zip › ijms-3542360-supplementary.pdf]
